# Supplementary material for: Cross-spectral fusion of thermal and RGB imaging for objective pain estimation
Source: PLOS Digit Health. 2026 May 15;5(5):e0001424. doi: 10.1371/journal.pdig.0001424 (PMC13178875; doi:10.1371/journal.pdig.0001424)
Supplement: S1 File — Contains the following components: Table A in S1 Appendix. State-of-the-Art Comparison for Automated Pain Recognition (2008–2025). Comprehensive comparison spanning traditional machine learning, deep learning (CNN, LSTM, Transformer), thermal imaging, and cross-spectral fusion methods (20 + entries) [40]. Moved from main text per Reviewer 1 recommendation. Table B in S1 Appendix. Complete Per-Fold 5-Fold Cross-Validation Results. Individual fold MAE, RMSE, PCC, ICC, and 3-class Accuracy for CSAF+Transformer and all baselines on Dataset 1 (controlled), Dataset 2 (clinical), and Combined datasets. Mean±std summaries and 95 % CI for primary metric. Referenced in Tables 2, 1 and Section 3. Table C in S1 Appendix. Extended Hardware Specifications, Camera Synchronisation Protocol, Pain Induction Protocol Rationale, and Environmental Control Justification. Moved from main Section 3.2 to reduce manuscript length per Reviewer 1 recommendation. Referenced in Fig 1 caption and Section 2. Table D in S1 Appendix. Fairness Audit — Subgroup Performance. CSAF+Transformer vs. RGB-Only MAE across age group, sex, Fitzpatrick skin type (I–VI), expression intensity, pain type (CPT / algometry / postoperative), and occlusion presence. Note: Race/ethnicity audit not available for current datasets (acknowledged as limitation). “Sedated” subgroup = pharmacologically reduced facial mobility in controlled settings, NOT ICU patients on mechanical ventilation. Referenced in Tables 4, 5. Table E in S1 Appendix. Per-Stage Parameter Count and Training Time Breakdown for CSAF+Transformer (87M parameters total), including 4×NVIDIA A100 GPU training times per stage and inference benchmarks. Referenced in Sections 2 and 3. S1 Algorithm. Spatial registration and ROI extraction — full detail. Complete pseudocode including checkerboard calibration protocol, homography computation, landmark-based face alignment, bounding box table, and thermal normalisation procedure. Extended version of Algorithm 1. S2 A [file pdig.0001424.s001.pdf]

Supporting Information — S1 Appendix

Cross-Spectral Fusion of Thermal and RGB Imaging for Objective Pain Estimation

Oussama El Othmani, Sami Naouali

PLOS Digital Health — PDIG-D-26-00203

List of Supporting Information

S1 Appendix. Supporting tables for *Cross-Spectral Fusion of Thermal and RGB Imaging for Objective Pain Estimation.* Contains Tables A–E and Algorithms S1–S2.

Table A in S1 Appendix. State-of-the-Art Comparison for Automated Pain Recognition (2008–2025). Comprehensive comparison spanning traditional machine learning, deep learning (CNN, LSTM, Transformer), thermal imaging, and cross-spectral fusion methods. Moved from main text Table 1 per Reviewer 1 recommendation.

Table B in S1 Appendix. Complete per-fold 5-fold cross-validation results for CSAF+Transformer and all baselines on Dataset 1 (controlled), Dataset 2 (clinical), and Combined datasets. Reports MAE, RMSE, PCC, ICC, and 3-class Accuracy for every fold individually, plus mean±std summaries.

Table C in S1 Appendix. Extended hardware specifications, camera synchronisation protocol, pain induction protocol rationale, and environmental control justification. Moved from main text Section 3.2 to reduce manuscript length per Reviewer 1 recommendation.

Table D in S1 Appendix. Fairness audit: subgroup performance across age group, sex, skin type (Fitzpatrick scale), expression intensity, pain type (CPT vs. algometry vs. postoperative), and occlusion presence. Note: race/ethnicity subgroup audit not available for the current datasets; this limitation is acknowledged in Section 6 (Discussion) of the main manuscript.

Table E in S1 Appendix. Per-stage parameter count breakdown for CSAF+Transformer (total 87M parameters), including stage-by-stage training time on 4×NVIDIA A100 GPUs.

S1 Algorithm. Spatial Registration and ROI Extraction — full detail including calibration checkerboard protocol, homography computation, and landmark-based face alignment (extended version of Algorithm 1 in main manuscript).

S2 Algorithm. Cross-Spectral Attention Fusion (CSAF) — full detail including bidirectional cross-attention, adaptive gating, and residual connection (extended version of Algorithm 2 in main manuscript).

Table A in S1 Appendix. State-of-the-Art Comparison for Automated Pain Recognition

Table A in S1 Appendix. State-of-the-Art Comparison for Automated Pain Recognition. Comprehensive comparison of existing methods across modality, architecture, dataset, and performance metric. Performance metrics vary by task formulation: accuracy (%) for classification, MAE for regression, mIoU for detection. Our work is the first to apply cross-spectral fusion (RGB+Thermal) specifically to *continuous* pain intensity estimation with clinical validation.

Method	Year	Modality	Architecture	Dataset	Performance
Traditional Machine Learning Era (2008–2011)					
Prkachin & Solomon [15]	2008	RGB	FACS+PSPI	Shoulder Pain	66% (manual AU)
Ashraf et al. [16]	2009	RGB	AAM+SVM	UNBC-McMaster	66.7% accuracy
Lucey et al. [17]	2011	RGB	AAM+SVM	UNBC-McMaster	68.3% accuracy
Deep Learning Revolution (2014–2021)					
Werner et al. [18]	2014	RGB	CNN	BioVid	71.2% accuracy
Rodriguez et al. [19]	2017	RGB	LSTM	UNBC-McMaster	75.8% accuracy
Thiam et al. [21]	2019	Multi	CNN+Physio	BioVid	73.4% (RGB+ECG)
Zhou et al. [20]	2021	RGB	R-CNN	UNBC-McMaster	MAE=1.45
Attention Mechanisms & Transformers (2022–2024)					
Tavakolian & Hadid [2]	2022	RGB	3D CNN+Att.	UNBC-McMaster	76.3% accuracy
Lin et al. [25]	2022	Multi	Decision Fusion	Custom (n=28)	78.5% (9 modalities)
Tavakolian et al. [3]	2023	RGB	Vision Transformer	Multiple	72–78% accuracy
Gkikas et al. [7]	2024	RGB+HR	Transformer	BioVid	82.74% binary
Gu et al. [5]	2024	Multi	Stat. Correlation	Survey	— (methodology)
Wang et al. [4]	2024	RGB	Various	Multiple	— (survey)
Khan et al. [22]	2025	Multi	Systematic Review	Multiple	— (review)
Thermal Imaging for Pain (2013–2024)					
Manini et al. [14]	2013	Thermal	Manual Inspection	ED Patients (n=20)	Qualitative
Kopaczka et al. [13]	2016	Thermal	SVM	Neonatal (n=26)	69.2% accuracy
Pirie et al. [6]	2024	Thermal	Deep Learning	Neonatal	74.2% binary
Martinez-Jimenez et al. [8]	2024	Thermal	Algorithm	Burn Patients	Clinical utility
Cross-Spectral Fusion (Non-Pain Applications)					
Liao et al. [23]	2022	RGB+Thermal	Fusion-Encoder	RGB-T SOD	mIoU: 0.891
Liang et al. [24]	2023	RGB+Thermal	Explicit Attention	Multiple Tasks	+1.6–8.1% gain
Li et al. [9]	2023	RGB+Thermal	Dual-Stream CNN	Stress Detection	15.8% improvement
**CSAF+Transformer (Ours)**	**2025**	**RGB+Thermal**	**Cross-Attn+Temporal**	**Pain** (n=80)	**MAE=0.87 (29.3%)**

Abbreviations: AAM=Active Appearance Model; AU=Action Unit; CNN=Convolutional Neural Network; ECG=Electrocardiography; ED=Emergency Department; FACS=Facial Action Coding System; HR=Heart Rate; LSTM=Long Short-Term Memory; MAE=Mean Absolute Error; mIoU=mean Intersection over Union; Multi=Multiple modalities; PSPI=Pained Structure of Pain Intensity; R-CNN=Recurrent CNN; RGB=Red-Green-Blue; SOD=Salient Object Detection; SVM=Support Vector Machine.

Table B in S1 Appendix. Complete Per-Fold 5-Fold Cross-Validation Results

Table B in S1 Appendix. Complete per-fold 5-fold cross-validation results for CSAF+Transformer on Dataset 1 (controlled), Dataset 2 (clinical), and the Combined dataset. Primary metric: MAE (Mean Absolute Error, lower is better). Secondary metrics: RMSE (Root Mean Square Error), PCC (Pearson Correlation Coefficient, higher is better), ICC (Intraclass Correlation Coefficient, higher is better), Acc (3-class classification accuracy, higher is better). All metrics computed on the held-out test fold only. Referenced in main manuscript Tables 2, 3 and Section 4.2.

Dataset	Fold	MAE↓	RMSE↓	PCC↑	ICC↑	Acc (%)↑
Dataset 1 (Controlled)	Fold 1	0.81	1.14	0.88	0.85	83.7
	Fold 2	0.76	1.09	0.89	0.86	84.2
	Fold 3	0.83	1.17	0.87	0.84	82.9
	Fold 4	0.78	1.11	0.88	0.85	83.5
	Fold 5	0.77	1.10	0.89	0.86	84.8
	**Mean±std**	$\mathbf{0.79 \pm 0.03}$	1.12 ± 0.03	0.88 ± 0.01	0.85 ± 0.01	83.8 ± 0.7
Dataset 2 (Clinical)	Fold 1	1.12	1.51	0.83	0.80	80.1
	Fold 2	1.05	1.44	0.84	0.81	81.3
	Fold 3	1.09	1.48	0.83	0.80	79.8
	Fold 4	1.11	1.49	0.84	0.81	80.6
	Fold 5	1.03	1.42	0.85	0.82	81.9
	**Mean±std**	$\mathbf{1.08 \pm 0.04}$	1.47 ± 0.04	0.84 ± 0.01	0.81 ± 0.01	80.7 ± 0.8
Combined	Fold 1	0.89	1.23	0.87	0.84	82.9
	Fold 2	0.83	1.17	0.88	0.85	83.5
	Fold 3	0.90	1.24	0.86	0.83	81.8
	Fold 4	0.88	1.22	0.87	0.84	82.4
	Fold 5	0.85	1.19	0.88	0.85	83.3
	**Mean±std**	$\mathbf{0.87 \pm 0.03}$	1.21 ± 0.03	0.87 ± 0.01	0.84 ± 0.01	82.8 ± 0.6

Abbreviations: MAE=Mean Absolute Error; RMSE=Root Mean Square Error; PCC=Pearson Correlation Coefficient; ICC=Intraclass Correlation Coefficient; Acc=3-class classification accuracy (Low 0–3 / Moderate 4–6 / High 7–10).

↓ lower is better; ↑ higher is better.

95% CI for combined-dataset MAE of CSAF+Transformer: $[0.83, 0.91]$ (bootstrap B=1000, pooled test predictions).

Stratified 5-fold: each fold maintains similar pain intensity distribution.

Table C in S1 Appendix. Extended Hardware Specifications, Synchronisation Protocol, and Experimental Rationale

C.1 — RGB Camera Justification

We selected a high-resolution DSLR (Canon EOS 90D, 1920×1080, 30 fps) based on the following evidence from prior pain recognition literature [4, 18, 20]:

- **Spatial resolution (1920×1080 vs. typical 640×480 webcam):** Higher resolution enables detection of subtle facial micro-expressions (Action Units AU4: brow lowerer; AU6/7: orbital tightening; AU9/10: levator contraction) that span only 5–10 pixels at lower resolution and are critical pain indicators [17].
- **Temporal resolution (30 fps):** Balances capturing rapid pain onset dynamics (expressions can change within 1–2 s for acute pain) with data storage and processing constraints [19].
- **Controlled LED lighting (5000 K, 800 lux):** Follows established protocols for standardised facial imaging [16], minimising shadow artefacts and ensuring consistent illumination across subjects and recording sessions.

C.2 — Thermal Camera Justification

We selected the FLIR A655sc thermal camera based on four critical factors derived from the thermal imaging pain literature:

1. **Thermal sensitivity (< 0.04°C at 30°C):** Pain-related temperature changes are often subtle (0.3–1.5°C per our findings). The A655sc's < 0.04°C sensitivity exceeds the 0.1°C threshold recommended for detecting autonomic responses to pain [11, 13]. Consumer-grade thermal cameras (sensitivity 0.1–0.2°C) may miss these signals; validation with affordable hardware is a stated future direction.
2. **Spatial resolution (640×480 pixels):** Sufficient to resolve individual facial ROIs (periorbital, nasal, forehead), each spanning approximately 80×80 to 120×120 pixels, enabling detection of localised vascular responses [12].
3. **Temporal resolution (30 fps):** Synchronized with RGB for precise temporal alignment, critical given our finding of 1.2±0.3 s thermal precedence before visible expressions. Lower frame rates (10–15 fps) could miss rapid autonomic responses.
4. **Spectral range (7.5–14 μm longwave infrared):** Captures skin surface temperature emissions without atmospheric absorption interference. Skin emissivity ≈ 0.98 in this range regardless of skin pigmentation, providing equitable performance across Fitzpatrick Types I–VI [10].

C.3 — Camera Synchronisation Protocol

Precise temporal alignment between thermal and RGB streams is critical for valid multimodal fusion. We implemented hardware-triggered synchronisation:

1. **Hardware Trigger:** A National Instruments USB-6001 DAQ sends simultaneous TTL pulses (5 V, 10 ms) to both cameras' external trigger inputs at 30 Hz.
2. **Timestamp Recording:** Each camera records its internal timestamp (microsecond precision) for every frame. Post-acquisition, synchronisation is verified by computing inter-camera timestamp offsets.
3. **Synchronisation Verification:** Median offset = 3.2 ms (IQR: 2.1–4.8 ms); maximum offset = 9.7 ms across all 105 h of recording. All frames meet the $<$10 ms criterion (30 fps = 33 ms per frame).

4. **Spatial Registration:** Homography transformation **H** computed from a calibration checkerboard (10×10 squares, 25 mm) visible in both spectra. Cameras separated by 15 cm horizontally to prevent RGB illumination interference with the thermal sensor.

5. **Validation:** An LED light source (visible to RGB; generates heat visible to thermal) pulsed at 1 Hz. Both cameras detected state transitions within the same 33 ms frame window (measured lag < 10 ms).

C.4 — Pain Induction Protocol Rationale

We employed CPT and pressure algometry as gold-standard experimental pain models:

- **Cold Pressor Test (CPT):** Induces gradually increasing ischaemic pain with well-characterised autonomic responses including sympathetic activation, peripheral vasoconstriction, increased heart rate and blood pressure, and cortisol release [1]. These autonomic changes manifest as measurable thermal signatures (skin cooling from vasoconstriction), making CPT ideal for validating thermal imaging sensitivity.
- **Pressure Algometry:** Provides localised mechanical nociceptive input with reproducible intensity control via calibrated pressure application (1–10 $\mathrm{kg/cm^2}$). Allows systematic variation across the pain scale while maintaining standardised stimulus [18].
- **Clinical Postoperative Data:** Provides essential real-world validation with expected higher inter-subject variability (different surgical procedures, analgesic regimens, individual sensitivities).

C.5 — Environmental Control

Temperature-controlled rooms (21–23°C, 40–60% humidity) are essential for thermal imaging validity [10]. Ambient temperature variations > 2°C can induce facial thermal changes (0.5–1.0°C) comparable to pain-related vascular responses, confounding analysis. Temperature was logged every 60 s throughout recording. Prior studies lacking environmental control [14] reported 40–60% higher variance in thermal measurements.

Table D in S1 Appendix. Fairness Audit: Subgroup Performance

Table D in S1 Appendix. Fairness audit: CSAF+Transformer performance across subgroups. Referenced in main manuscript Tables 5 and 6 and Section 4.8. **Note:** Race/ethnicity subgroup audit is not available for the current datasets; this limitation is acknowledged in Section 6 (Discussion) of the main manuscript and constitutes a priority for future multi-centre data collection. **"Sedated" subgroup (n=12):** controlled-setting subjects with pharmacologically reduced facial mobility (midazolam), NOT mechanically ventilated ICU patients.

**Attribute**	**Subgroup**	n (subjects)	**RGB-Only MAE**	**CSAF MAE**	**Impr.(%)**
Age group	Young (< 40 yr)	24	1.12 ± 0.12	0.81 ± 0.08	27.7
	Middle (40–64 yr)	38	1.21 ± 0.11	0.86 ± 0.08	28.9
	Elderly ($\geq$ 65 yr)	18	1.42 ± 0.16	1.03 ± 0.11	27.5
Sex	Female	42	1.20 ± 0.10	0.86 ± 0.07	28.3
	Male	38	1.27 ± 0.11	0.89 ± 0.08	29.9
Skin tone (Fitzpatrick)	Type I–II (Light)	28	1.15 ± 0.13	0.83 ± 0.09	27.8
	Type III–IV (Medium)	38	1.24 ± 0.11	0.87 ± 0.08	29.8
	Type V–VI (Dark)	14	1.38 ± 0.17	0.92 ± 0.12	33.3
Expression level	High expressers (top 25%)	20	1.02 ± 0.09	0.76 ± 0.07	25.5
	Low expressers (bottom 25%)	20	1.57 ± 0.21	0.98 ± 0.14	37.6
Pain type	CPT (cold pressor)	50	1.19 ± 0.10	0.83 ± 0.07	30.2
	Algometry (pressure)	50	1.26 ± 0.11	0.88 ± 0.08	30.2
	Postoperative (surgical)	30	1.51 ± 0.15	1.08 ± 0.11	28.5
Facial occlusion	No occlusion	68	1.18 ± 0.10	0.84 ± 0.07	28.8
	Partial occlusion (mask/hand)	12	1.52 ± 0.19	1.06 ± 0.15	30.3
Dataset	Controlled (Dataset 1)	50	1.18 ± 0.10	0.79 ± 0.06	33.1
	Clinical (Dataset 2)	30	1.51 ± 0.14	1.08 ± 0.10	28.5

Abbreviations: MAE=Mean Absolute Error; Impr.=improvement over RGB-Only; CPT=Cold Pressor Test.
Improvement: (RGB-Only MAE − CSAF MAE) / RGB-Only MAE $\times$100%.
All improvements significant (p<0.01, paired t-test, Bonferroni corrected).
Race/ethnicity subgroup audit not available for current datasets. Dataset does include diverse skin tones (Fitzpatrick I–VI) but race/ethnicity self-identification was not collected.

Table E in S1 Appendix. Per-Stage Parameter Count and Training Time Breakdown

Table E in S1 Appendix. Per-stage parameter count for CSAF+Transformer (total 87 million trainable parameters) and training time on 4×NVIDIA A100 (40 GB) GPUs using PyTorch 2.0.1 DistributedDataParallel. Inference: 38 ms/frame on GPU (26.3 fps); 182 ms/frame on CPU (5.5 fps). Model checkpoint: 332 MB (FP32); 166 MB (FP16, $<$ 0.02 MAE increase on validation set). Referenced in main manuscript Sections 3.6 and 4.1.

Module	Parameters (M)	Stage Trained	Epochs	Training Time
RGB Encoder (ResNet-50, pretrained ImageNet+VGGFace2)	25.6	Stage 1	20	11.5 h
Thermal Encoder (ResNet-50, trained from scratch)	25.6	Stage 1	20	(included above)
CSAF Fusion Modules (5 ROIs × bidirectional attention)	24.1	Stage 2	30	14.2 h
Temporal Transformer (6 layers, 8 heads, d=512)	11.0	Stage 3	50	
Regression Head (2×512 → 1)	0.7	Stage 3	50	(included)
**Total**	**87.0**	**3 stages**	**100**	**≈48 h total**

Abbreviations: M=millions; FP16=16-bit float; FP32=32-bit float; GPU=Graphics Processing Unit; ROI=Region of Interest; h=hours.

Stage 3 (end-to-end fine-tuning, 50 epochs) ran for approximately 22.3 h.

Batch size: 16 (300-frame sequences). AdamW optimizer; lr=10^{-4}, cosine annealing; gradient clipping (max norm 1.0).

The PatchGAN discriminator (if used in future domain-adaptation extensions) is training-only and not loaded at inference.

S1 Algorithm. Spatial Registration and ROI Extraction (Full Detail)

Algorithm 1 Spatial Registration and ROI Extraction

Require: RGB image $I_{rgb} \in \mathbb{R}^{H \times W \times 3}$, Thermal image $I_{th} \in \mathbb{R}^{H_{th} \times W_{th}}$, Pre-computed homography $\mathbf{H} \in \mathbb{R}^{3 \times 3}$ (from checkerboard calibration), Canonical face landmark template $\{x_i^{canonical}\}_{i=1}^{5}$

Ensure: Aligned thermal image $I_{th}^{aligned}$, 5 ROI pairs $\{R_{rgb}^r, R_{th}^r\}_{r=1}^5$ each at 128×128 px

Step 1: Thermal-RGB spatial alignment
$I_{th}^{aligned} \leftarrow \text{ApplyHomography}(I_{th}, \mathbf{H})$ ▷ Warp thermal to RGB coordinate frame ▷ Registration error: mean 1.8 px across dataset
Step 2: Face detection (RGB)
$\{B_{face}, \{x_i\}_{i=1}^5\} \leftarrow \text{RetinaFace}(I_{rgb})$ ▷ 99.1% detection rate; 5 landmarks: eyes, nose, mouth corners
if no face detected **then**
  return null ▷ Frame discarded; $< 0.9\%$ of all frames
end if
Step 3: Similarity transform alignment
$\mathbf{T} \leftarrow \text{EstimateSimilarity}(\{x_i\}, \{x_i^{canonical}\})$ ▷ Least-squares; corrects scale, rotation, translation
$I_{rgb}^{aligned} \leftarrow \text{WarpAffine}(I_{rgb}, \mathbf{T}, (512, 512))$
$I_{th}^{aligned} \leftarrow \text{WarpAffine}(I_{th}^{aligned}, \mathbf{T}, (512, 512))$
Step 4: ROI extraction
for $r = 1$ to 5 **do** ▷ ROIs: 1=Periorbital, 2=Forehead, 3=Nasal, 4=Cheeks, 5=Perioral
  $R_{rgb}^r \leftarrow \text{CropResize}(I_{rgb}^{aligned}, bbox_r, 128{\times}128)$ ▷ Pain-relevant anatomy per [15]
  $R_{th}^r \leftarrow \text{CropResize}(I_{th}^{aligned}, bbox_r, 128{\times}128)$
end for
Step 5: Thermal normalisation
for $r = 1$ to 5 **do**
  $R_{th}^r \leftarrow \frac{R_{th}^r - \mu_{baseline}^r}{\sigma_{baseline}^r}$ ▷ Normalise by 3-min baseline mean/std per ROI
end for
return $I_{th}^{aligned}, \{R_{rgb}^r, R_{th}^r\}_{r=1}^5$

Bounding Box Definitions. ROI bounding boxes ($bbox_r$) in canonical 512×512 face space:

ROI	Anatomical Region	$\mathbf{x_{min}}$	$\mathbf{y_{min}}$	$\mathbf{x_{max}}$	$\mathbf{y_{max}}$
1	Periorbital (eyes, AU6/7)	80	140	432	260
2	Forehead (brow, AU4)	100	60	412	155
3	Nasal (nose, perinasal)	170	240	342	370
4	Cheeks (bilateral zygomatic)	50	250	462	380
5	Perioral (mouth, AU9/10)	130	350	382	460

S2 Algorithm. Cross-Spectral Attention Fusion (CSAF) — Full Detail

Algorithm 2 Cross-Spectral Attention Fusion (CSAF)

Require: RGB regional features $\{F_{rgb}^r\}_{r=1}^5$, $F_{rgb}^r \in \mathbb{R}^{2048}$ from ResNet-50 global average pool
Thermal regional features $\{F_{th}^r\}_{r=1}^5$, $F_{th}^r \in \mathbb{R}^{2048}$
Ensure: Fused features $\{F_{fused}^r\}_{r=1}^5$, Attention maps $\{\alpha_{rgb\leftarrow th}^r, \alpha_{th\leftarrow rgb}^r\}_{r=1}^5$
Initialise learnable projection matrices:
$W_Q^{rgb}, W_K^{rgb}, W_V^{rgb}, W_Q^{th}, W_K^{th}, W_V^{th} \in \mathbb{R}^{2048\times 2048}$; $d_k = 2048$
$W_{gate} \in \mathbb{R}^{4096\times 2}$; $b_{gate} \in \mathbb{R}^2$
for $r = 1$ to 5 **do** ▷ Process each of 5 facial ROIs
— **Thermal-to-RGB cross-attention** —
$Q_{rgb} \leftarrow W_Q^{rgb} F_{rgb}^r$
$K_{th} \leftarrow W_K^{th} F_{th}^r$
$V_{th} \leftarrow W_V^{th} F_{th}^r$
$e_{rgb\leftarrow th} \leftarrow Q_{rgb} K_{th}^T / \sqrt{d_k}$ ▷ Scaled dot-product scores
$\alpha_{rgb\leftarrow th}^r \leftarrow \mathrm{softmax}(e_{rgb\leftarrow th})$ ▷ Attention weights: which thermal dims inform RGB
$F_{rgb\leftarrow th}^r \leftarrow \alpha_{rgb\leftarrow th}^r V_{th}$ ▷ Thermally-attended RGB features
— **RGB-to-Thermal cross-attention** —
$Q_{th} \leftarrow W_Q^{th} F_{th}^r$
$K_{rgb} \leftarrow W_K^{rgb} F_{rgb}^r$
$V_{rgb} \leftarrow W_V^{rgb} F_{rgb}^r$
$e_{th\leftarrow rgb} \leftarrow Q_{th} K_{rgb}^T / \sqrt{d_k}$
$\alpha_{th\leftarrow rgb}^r \leftarrow \mathrm{softmax}(e_{th\leftarrow rgb})$ ▷ Attention weights: which RGB dims inform thermal
$F_{th\leftarrow rgb}^r \leftarrow \alpha_{th\leftarrow rgb}^r V_{rgb}$
— **Adaptive gating** —
$[\lambda_{rgb}^r, \lambda_{th}^r] \leftarrow \sigma(W_{gate}[F_{rgb}^r; F_{th}^r] + b_{gate})$ ▷ σ=sigmoid; $\lambda \in [0,1]$ ▷ Learned per-ROI modality contribution weights
— **Residual fusion** —
$F_{fused}^r \leftarrow F_{rgb}^r + \lambda_{rgb}^r \cdot F_{rgb\leftarrow th}^r + \lambda_{th}^r \cdot F_{th\leftarrow rgb}^r$ ▷ Residual connection preserves unimodal features
end for
return $\{F_{fused}^r\}_{r=1}^5$, $\{\alpha_{rgb\leftarrow th}^r, \alpha_{th\leftarrow rgb}^r\}_{r=1}^5$

▷ **Implementation notes:** ▷ – All projections initialised with Xavier uniform ▷ – Dropout p=0.1 applied to attention weights during training ▷ – Gradient flow: both λ branches are differentiable; no stop-gradient ▷ – Memory: 5 ROIs × 2048^2 float32 ≈ 160 MB per batch item ▷ – At test time, λ_{rgb}^r and λ_{th}^r are logged per-frame for interpretability analyses (Table D in S1 Appendix and main Tables 3, 5)

References

References

[1] Mourot L, Bouhaddi M, Regnard J. Effects of the cold pressor test on cardiac autonomic control in normal subjects. *Physiological Research*. 2009;58(1):83–91.

[2] Tavakolian M, Hadid A. Deep spatiotemporal representation of the face for automatic pain intensity estimation. *Image and Vision Computing*. 2022;120:104402. doi:10.1016/j.imavis.2022.104402

[3] Tavakolian M, Hadid A, Abreu JG. Vision transformers for pain detection. *IEEE Access*. 2023;11:17472–17483. doi:10.1109/ACCESS.2023.3248129

[4] Wang S, Li Y, Zhang H. Automatic Recognition Methods Supporting Pain Assessment: A Survey. *IEEE Transactions on Affective Computing*. 2024;15(1):1–20. doi:10.1109/TAFFC.2020.3023604

[5] Gu X, Wang Z, Jin I, Wu Z. Advancing multimodal data fusion in pain recognition. *Multimodal Technologies and Interaction*. 2024;8(3):18. doi:10.3390/mti8030018

[6] Pirie C, Ferreira LA, Coutrin GAS, Carlini LP, Quinlan R. Explaining and upsampling for neonatal pain assessment. arXiv:2407.15865. 2024.

[7] Gkikas S, Tachos N, Andreadis S, Pezoulas V, Zaridis D, Gkois G, et al. Multimodal automatic assessment of acute pain through facial videos and heart rate signals. *Frontiers in Pain Research*. 2024;4:1372814. doi:10.3389/fpain.2024.1372814

[8] Martinez-Jimenez MA, Ramirez-GarciaLuna JL, Kolosovas-Machuca ES. Development and validation of an algorithm for automated pain management in medicine. *Burns*. 2024;50(4):892–901. doi:10.1016/j.burns.2024.02.008

[9] Li X, Wang Y, Zhang P, Hou Y, Hu B. RGB and Infrared Fusion for Stress Detection Using Dual-Stream CNN. *Sensors*. 2023;23(15):6789. doi:10.3390/s23156789

[10] Lahiri BB, Bagavathiappan S, Jayakumar T, Philip J. Medical applications of infrared thermography: A review. *Infrared Physics & Technology*. 2012;55(4):221–235. doi:10.1016/j.infrared.2012.03.007

[11] Ioannou S, Gallese V, Merla A. Thermal infrared imaging in psychophysiology: Potentialities and limits. *Psychophysiology*. 2014;51(10):951–963. doi:10.1111/psyp.12243

[12] Drummond PD, Lance JW. Facial temperature in migraine, tension-type, and cervicogenic headache. *Headache*. 1984;24(2):59–68.

[13] Kopaczka M, Kolk R, Merhof D. A fully annotated thermal face database and its application for thermal facial expression recognition. In: *Proc. IEEE I2MTC*. 2016. p.1–6. doi:10.1109/I2MTC.2016.7520410

[14] Manini AF, Fleischman RJ, Olson D, Zaso MJ, Carbonaro T. Feasibility of infrared thermal imaging for the assessment of acute pain in the emergency department. *Academic Emergency Medicine*. 2013;20:S266.

[15] Prkachin KM, Solomon PE. The structure, reliability and validity of pain expression: Evidence from patients with shoulder pain. *Pain*. 2008;139(2):267–274. doi:10.1016/j.pain.2008.04.010

[16] Ashraf AB, Lucey S, Cohn JF, Chen T, Ambadar Z, Prkachin KM, Solomon PE. The painful face–pain expression recognition using active appearance models. *Image and Vision Computing*. 2009;27(12):1788–1796. doi:10.1016/j.imavis.2009.05.007

[17] Lucey P, Cohn JF, Prkachin KM, Solomon PE, Matthews I. Painful data: The UNBC-McMaster shoulder pain expression archive database. In: *Proc. IEEE FG*. 2011. p.57–64. doi:10.1109/FG.2011.5771462

[18] Werner P, Al-Hamadi A, Niese R, Walter S, Gruss S, Traue HC. Automatic Pain Recognition from Video and Biomedical Signals. In: *Proc. ICPR.* 2014. p.4582–4587. doi:10.1109/ICPR.2014.783

[19] Rodriguez P, Cucurull G, Gonzalez J, Gonfaus JM, Nasrollahi K, Moeslund TB, Roca FX. Deep pain: Exploiting long short-term memory networks for facial expression classification. *IEEE Transactions on Cybernetics.* 2022;52(6):3314–3324. doi:10.1109/TCYB.2017.2662199

[20] Zhou J, Hong X, Su F, Zhao G. Recurrent convolutional neural network regression for continuous pain intensity estimation in video. *IEEE Transactions on Circuits and Systems for Video Technology.* 2021;31(3):1152–1165. doi:10.1109/TCSVT.2020.2999999

[21] Thiam P, Bellmann P, Kestler HA, Schwenker F. Exploring deep physiological models for nociceptive pain recognition. *Sensors.* 2019;19(20):4503. doi:10.3390/s19204503

[22] Khan MU, Chetty G, Goecke R, Fernandez-Rojas R. A Systematic Review of Multimodal Signal Fusion for Acute Pain Assessment Systems. *ACM Computing Surveys.* 2025;57(3):1–37. doi:10.1145/3737281

[23] Liao G, Gao W, Li G, Wang J, Kwong S. Cross-Collaborative Fusion-Encoder Network for Robust RGB-Thermal Salient Object Detection. *IEEE Transactions on Circuits and Systems for Video Technology.* 2022;33(1):430–443. doi:10.1109/TCSVT.2022.3184840

[24] Liang M, Hu J, Bao C, Feng H, Deng F, Lam TL. Explicit Attention-Enhanced Fusion for RGB-Thermal Perception Tasks. *IEEE Robotics and Automation Letters.* 2023;8(7):4060–4067. doi:10.1109/LRA.2023.3272269

[25] Lin YY, Xiao Y, Wang L, Guo Y, Zhu W, Dalip B, et al. Experimental Exploration of Objective Human Pain Assessment Using Multimodal Sensing Signals. *Frontiers in Neuroscience.* 2022;16:831627. doi:10.3389/fnins.2022.831627
