# Supplementary material for: Cross-spectral fusion of thermal and RGB imaging for objective pain estimation
Source: PLOS Digit Health. 2026 May 15;5(5):e0001424. doi: 10.1371/journal.pdig.0001424 (PMC13178875; doi:10.1371/journal.pdig.0001424)
Supplement: Attachment — Submitted filename: Response_to_Reviewers.pdf [file pdig.0001424.s003.pdf]

Response to Reviewers

PDIG-D-26-00203

Cross-Spectral Fusion of Thermal and RGB Imaging for Objective Pain Estimation

PLOS Digital Health

Submitted: March 2026 | Corresponding Author: Sami Naouali (salnawali@kfu.edu.sa)

We sincerely thank the Academic Editor (Dr. Jia-Lang Xu), Section Editor (Dr. Henry Horng-Shing Lu), Editor-in-Chief (Dr. Leo Anthony Celi), and both reviewers (Dr. Philip Chung and Dr. Pei-Chun Lin) for their thorough and constructive evaluation of our manuscript. We have carefully addressed every point raised. Below, each reviewer comment is quoted verbatim in italics, followed by our detailed response in blue. All changes in the manuscript are marked with **\rev{...}** (new/revised text, shown in blue) and **\del{...}** (deleted text, shown in red strikethrough) in the "Revised Manuscript with Track Changes" file. Reviewer-specific editorial notes are shown in teal **\rvcomment{...}** throughout the revision.

JOURNAL REQUIREMENTS (7 items)

Requirement 1 — Ethics Statement location

Please ensure that your Ethics Statement is available in its entirety at the beginning of your Methods section, under a subheading 'Ethics Statement'.

Done. The Ethics Statement (previously located in the Declarations section at the end of the manuscript) has been moved verbatim to the opening of Section 2 (Methods) under its own \subsection*{Ethics Statement} heading. The full text of the IRB approval (Protocol #2023-041-PAIN), Declaration of Helsinki compliance, informed consent procedures, anonymization, and data-deletion protocols is now present at the start of the Methods, as required. The Declarations section retains only the conflict-of-interest, contributions, and funding statements.

Requirement 2 — Manuscript source file format

We ask that a manuscript source file is provided at Revision. Please upload your manuscript file as a .doc, .docx, .rtf or .tex.

We submit a single self-contained .tex file (Manuscript.tex). The bibliography has been embedded directly as a \begin{thebibliography}{99} ... \end{thebibliography} environment within the .tex file, eliminating the need for a separate .bib file or external bibliography style file. All 38 references are preserved in full.

Requirement 3 — Author Summary (150–200 words)

Please provide an Author Summary. This should appear in your manuscript between the Abstract (if applicable) and the Introduction, and should be 150–200 words long.

Added. A 178-word Author Summary now appears immediately after the Abstract and before Section 1 (Introduction), formatted under the heading \section*{Author Summary}. The summary explains in plain language: (1) the clinical problem of pain assessment for non-verbal patients; (2) the dual-camera thermal+RGB approach; (3) the key finding of 29% improvement; and (4)

the 1-second thermal precedence result. It is written in first person and accessible to non-specialist readers, in line with PLOS Digital Health guidelines.

Requirement 4 — In-text citations and legends for Figures 12, 13, 14

Please ensure that all Figure files have corresponding citations and legends within the manuscript. Currently, Figures 12, 13, and 14 in your submission file inventory does not have an in-text citation.

Resolved as follows:

- Figure 14 (Human Expert Comparison): Now cited and fully captioned in Section 4.11 (Human Expert Comparison — new expanded sub-section). The caption details: sample size (n=200), stratification by pain intensity, expert credentials (8/11/15 years ICU experience), metrics shown (MAE, PCC, ICC), and the important caveat regarding information asymmetry between experts and the automated system.
- After careful review, All Figures not in the Clean Version they have been permanently removed from the file inventory. No content was lost.

Requirement 5 — Supporting Information legends

We have noticed that you have uploaded Supporting Information files, but you have not included a list of legends. Please add a full list of legends for your Supporting Information files after the references list.

A \section*{Supporting Information} block with full legends for S1 Table through S3 Table has been added immediately after the \end{thebibliography} in the manuscript source. A separate Supporting_Information.tex file is also submitted containing the full content of each supporting table with complete captions and footnotes. Legends follow PLOS formatting: bold label (e.g., S1 Table.) followed by title and descriptive caption.

Requirement 6 — Data Availability compliance

All PLOS journals now require all data underlying the findings described in their manuscript to be freely available to other researchers, either in a public repository, within the manuscript itself, or uploaded as supplementary information.

We have restructured our data availability to achieve the maximum possible compliance:

- Code and Model Weights: Fully publicly available without restriction at https://github.com/oussama123-ai/Cross-Spectral-Fusion-of-Thermal. No login or data-use agreement required.
- Anonymized Processed Features, Metadata (aggregated demographics, pain intensity distributions), and Evaluation Protocol (train/validation/test splits, cross-validation fold definitions, evaluation scripts): Deposited as Supporting Information files (S1–S3) freely downloadable with the published article — no restrictions.
- Raw Data Sample: A representative sample of anonymized raw data underlying this study is publicly deposited at: https://zenodo.org/records/18991937 (DOI: 10.5281/zenodo.18991937). This deposit includes example feature arrays to substantiate the processed features described in S2--S3, in compliance with PLOS open data requirements.
- Restricted Data: Full raw video recordings cannot be publicly released due to patient privacy protections and institutional review board (IRB) restrictions. Qualified researchers may request access to additional processed features by contacting the corresponding author with a data use agreement detailing intended use, privacy safeguards, and commitment to non-re-identification. Requests are reviewed within 30 days.

The Data and Code Availability statement in the manuscript has been updated to reflect these

three tiers clearly.

Requirement 7 — Figure 1 copyright (clip-art / icons)

Please confirm whether you drew the images / clip-art within the figure panels by hand. If you did not draw the images, please provide a link to the source of the images or icons and their license / terms of use; or written permission from the copyright holder.

Confirmed. All images, diagrams, icons, and clip-art elements within Figure 1 (System Architecture Pipeline) were drawn entirely by hand by the authors using vector graphics software. No third-party images, stock art, icon libraries, or copyrighted material from external sources were used in any figure panel. Figure 1 is therefore entirely original author work, fully compatible with the Creative Commons Attribution 4.0 International (CC BY 4.0) license required by PLOS journals. This confirmation has also been sent by separate reply email to the journal office.

All figure elements are original illustrations created by the authors (Wikimedia Commons: https://commons.wikimedia.org/wiki/File:Figure1-13-03-2026.tif, CC BY 4.0).

ADDITIONAL EDITOR COMMENTS

Editor Comment

This manuscript has been under review for a relatively long time, and we hope this delay has not affected your willingness to submit to this journal. Two reviewers have already provided their comments. Please respond to the issues raised by the reviewers.

We sincerely apologize for any inconvenience caused by the timeline and thank the editorial team for their patience and continued support. Our enthusiasm for publishing in PLOS Digital Health has not diminished. We have comprehensively addressed all comments from both reviewers as detailed below. The manuscript has been substantially restructured to meet PLOS standards and to address all technical concerns.

REVIEWER #1 — Philip Chung

Overview Comment

Authors have developed a new cross-attention fusion model using thermal imaging and RBG facial analysis and have conducted convincing experiments on the approach with two in-human datasets. This work is a good initial basic science/feasibility work. As authors mention, further validation is needed for target applications in neonates, cognitively impaired individuals, and sedated patients. My main concern is that the manuscript is too verbose and should be written in a more focused manner that more clearly conveys the authors' modeling and experimental contributions.

We thank Reviewer 1 for the thorough and constructive evaluation and for recognizing the scientific merit of the work. We have addressed the verbosity concern comprehensively: the revised manuscript is approximately 7,200 words (main text, excluding references, tables, figure captions, and supplementary material), reduced from ~13,000 words. This was achieved through the structural changes detailed in the major comments below, without removing any scientific content — all details are preserved in Supporting Information.

Major Comments

Major Comment 1 — Manuscript length (~13,000 words)

This manuscript is too long and reads more like a textbook chapter or review article rather than an original investigation research manuscript. It is roughly 13,000 words whereas most research manuscripts are typically in the range of 3,000–6,000 words. Almost every section contains too much detail. Same/similar information is restated across different sections. Excessive background detail buries the research team's main experimental contributions. Consider splitting some of the manuscript's elaboration on historical background, implementation details such as technical constraints, deployment barriers, discussion, etc. into a supplementary appendix.

Fully addressed. The revised main manuscript is approximately 7,200 words. The following content was moved to Supporting Information (S3 Table / supplementary appendix) rather than deleted, preserving all scientific detail:

- Full hardware justification paragraphs for RGB camera (Canon EOS 90D) and thermal camera (FLIR A655sc), including NEDT thresholds, spatial resolution rationale, and temporal resolution justification.
- Complete camera synchronization protocol (NI USB-6001 DAQ, TTL trigger timing, timestamp verification procedure, LED validation test).
- Pain induction protocol rationale (CPT and pressure algometry justifications).
- Environmental control rationale (temperature/humidity specifications and confound analysis).
- Extended clinical deployment barriers sub-section (all 5 barriers: regulatory, EHR integration, alarm fatigue, cost-effectiveness, training).
- Extended statistical considerations sub-section (multiple comparisons, overfitting risk analysis).
- Repetitive restatements of results across Introduction/Contributions/Results sections were consolidated.

Major Comment 2 — Introduction too long (4 pages)

The introduction/background/research vision/contributions is also too long spanning 4 pages (many research articles are only 4–6 pages in length). Please significantly shorten this to less than one page, focused around the research vision and hypothesis. The contributions/significance subsection ends up repeating the results section. Only briefly mention contributions at the end of the introduction section or move to the results section entirely.

Addressed. The Introduction has been restructured to approximately 1.5 pages covering three focused elements: (1) the clinical problem and motivation for automated pain assessment in non-verbal patients, (2) the gap in existing RGB-only automated approaches, and (3) our hypothesis and four high-level contributions in a concise bullet paragraph. Specifically:

- The Background and Motivation sub-section has been condensed from ~3 pages to 2 short paragraphs.
- The detailed wearables-vs-thermal comparison has been condensed to 2 sentences with citations.
- The detailed observational tools discussion (FLACC, NIPS, PAINAD specifics) has been condensed to 1 sentence.
- The Research Vision subsection has been absorbed into the Introduction's closing paragraph.
- The Contributions subsection no longer pre-states numerical results — it lists 4 methodological contributions only. Results are presented in the Results section.
- Paper Organization subsection has been removed entirely.
- The standalone sub-section headings (Background, Research Vision, Contributions, Organization) have been removed; the Introduction is now a unified narrative.

Major Comment 3 — Related Work section not appropriate for original research

The related work section needs to either be incorporated into introduction or discussion as it is not one of the sections of manuscript organization. This section makes the manuscript read more like a review article rather than a manuscript focused on new research methodology.

Addressed. The standalone Related Work section has been removed from the main manuscript. Its content has been redistributed as follows:

- A concise prior-work paragraph (6 sentences) has been integrated into the Methods section as context for our architectural choices, covering the trajectory from AAM+SVM (66-70% accuracy) through CNNs (70-75%), LSTMs (75-78%), and transformers (78-83%), and noting that all are RGB-only.
- The thermal imaging in medicine and multimodal fusion sub-sections have been condensed and integrated into the Discussion (Comparison to Prior Work sub-section).
- The Research Gaps sub-section content has been absorbed into the Introduction's motivation paragraph.
- The full state-of-the-art comparison Table 1 has been moved to Supporting Information (S1 Table) per Major Comment 4.

Major Comment 4 — Table 1 (prior research comparison) should be supplemental

Your Table 1 is a comparison against prior research. This is appropriate for a review article but not for a manuscript focusing on original research investigation. If you want to include this table, make it a supplemental table that summarizes existing literature. Commonly, Table 1 for original research articles in clinical medicine is often part of the results and includes a description of dataset/research cohort feature & outcome variables as well as patient population factors.

Done. The state-of-the-art comparison table has been moved to Supporting Information (S1 Table). Table 1 in the main manuscript is now the Dataset Statistics table (previously Table 2) describing participant demographics, recording statistics, pain intensity distributions, and per-fold sample counts. This is consistent with clinical research manuscript conventions as noted by the reviewer. The full SoTA comparison with all 20+ methods remains fully accessible in S1 Table.

Major Comment 5 — Cross-dataset generalization and human expert comparison deserve more detail

Authors summarize additional analyses on cross-dataset generalization and human expert comparison, but do not report details. These are two very important areas of results that affect the likelihood of actual clinical use & clinical adoption and should be included/emphasized in greater detail rather than an afterthought.

Both analyses have been substantially expanded into dedicated sub-sections in the Results:

- Section 5.9 (Cross-Dataset Generalization — NEW): Reports the complete zero-shot transfer result (models trained on Dataset 1, tested on Dataset 2: MAE=1.34, 95% CI [1.28, 1.40]); fine-tuning with 20% clinical data (MAE=1.15, $p<0.001$ vs zero-shot); in-distribution clinical performance (MAE=1.08). Discusses the domain gap sources: medication effects on autonomic tone, medical equipment occlusions, ambient temperature variability in PACU, and higher inter-patient variability in surgical pain. Recommends domain-adaptive fine-tuning for clinical deployment.
- Section 5.10 (Human Expert Comparison — EXPANDED): Now fully reports expert demographics (3 ICU nurses, 8, 11, and 15 years experience); evaluation subset design (n=200 samples, stratified: 67 low/67 moderate/66 severe); per-expert MAE, PCC, ICC with 95% CI (bootstrap B=1000); expert mean vs system comparison table (Table 5). Explicitly states and discusses two important caveats: (a) experts assessed RGB video only while our system used both modalities, making the comparison not perfectly symmetric; (b) experts

have access to additional clinical context unavailable to the vision system. Concludes that the comparison demonstrates approximate parity in controlled evaluation conditions, not equivalent real-world performance.

Minor Comments

Minor Comment 1 — Abstract combines two distinct datasets

Wording combines the 2 separate datasets for controlled pain induction vs. clinical postoperative monitoring, which are different. I would not combine these as they test different mechanisms/categories in pain research (induced discomfort vs. surgical pain).

The Abstract has been revised to clearly distinguish the two datasets from the outset. The revised abstract now introduces: Dataset 1 (controlled laboratory pain induction, Cold Pressor Test and pressure algometry, n=50 healthy adults, 87.3 h) explicitly framed as proof-of-concept validation; and Dataset 2 (real-world clinical postoperative monitoring, n=30 surgical patients, 17.7 h) explicitly framed as clinical generalization testing. Performance figures are reported separately in the Results (Section 5.2: Dataset 1 MAE=0.79, 33.1% improvement; Dataset 2 MAE=1.08, 28.5% improvement), and the combined figure (MAE=0.87, 29.3%) is retained as an overall summary with the distinction clearly noted.

Minor Comment 2 — Table 1 as supplemental (also in Major Comment 4)

Your Table 1 is a comparison against prior research. This is appropriate for a review article but not for a manuscript focusing on original research methodology. If you want to include this table, make it a supplemental table that summarizes existing literature.

Addressed — see Major Comment 4 response above. Table 1 in the revised manuscript is now the Dataset Statistics table. The SoTA comparison is S1 Table in Supporting Information.

Minor Comment 3 — Abbreviation 'NRS' unexplained in Table 2 caption

Explain abbreviation such as 'NRS' in captions.

All abbreviations (NRS = Numeric Rating Scale; VAS = Visual Analog Scale; CPT = Cold Pressor Test; IRB = Institutional Review Board; ICC = Intraclass Correlation Coefficient; PCC = Pearson Correlation Coefficient; MAE = Mean Absolute Error; AU = Action Unit; PACU = Post-Anesthesia Care Unit) are now defined on first use in the main text and are included in the Abbreviations footnotes of all tables and figure captions where they first appear. The Dataset Statistics table caption now includes a footnote: 'NRS = Numeric Rating Scale (0 = no pain, 10 = worst imaginable pain).' The Abbreviations section at the end of the manuscript lists all 26 abbreviations used.

Minor Comment 4 — Table 4 and Figure 3 show the same information; add error bars

Table 4 and Figure 3 mostly show the same information–please consolidate. If you choose to keep the plots in Figure 3, please add error bars/confidence intervals.

The redundancy has been resolved. We retained the stratified performance figure (Fig. 2 in revised numbering) as the primary visual presentation of the intensity-stratified results, since the figure conveys the monotonically increasing benefit across pain levels more intuitively than a table. The corresponding table has been removed from the main manuscript. All bar charts in the figure now include 95% confidence interval error bars computed via bootstrap resampling (B=1000) across the 5 cross-validation folds. The figure caption explicitly states: 'Error bars show 95% CI across 5 cross-validation folds.'

Minor Comment 5 — Designate a primary metric

Consider selecting one of the metrics as your 'primary' metric and the others as 'secondary' to help readers focus on a main metric for comparison.

MAE (Mean Absolute Error) is now designated as the primary outcome metric throughout the manuscript. This designation is established in Section 4.2 (Evaluation Metrics) with explicit justification: (1) our task is continuous regression on the 0–10 NRS scale, not classification; (2) MAE has direct clinical interpretability (MAE=0.87 corresponds to <1 NRS point average error, below the 1.5-point minimal clinically important difference); (3) MAE is the standard primary metric in continuous pain regression literature (Zhou et al. 2021; Gkikas et al. 2024; Khan et al. 2025). PCC, ICC, and 3-class accuracy are consistently labeled 'secondary metrics' in all tables, figures, and text throughout the manuscript.

Minor Comment 6 — Sedated/low-expressor populations: out-of-domain concern

The focus of this paper is to analyze and validate cross-modal fusion thermal + RBG deep learning models. Authors then suggest that the technology could be useful for low expressors or patients who are sedated where patients have limited facial change and presumably may have thermal changes, but this data distribution is out-of-domain compared to the examined datasets the authors study.

We fully agree with this assessment and have clarified throughout the manuscript. In Section 5.7 (Performance on Special Populations) and the Discussion, we now explicitly state: the 'sedated' subgroup in our study consists of controlled-setting subjects with pharmacologically reduced facial mobility (induced expression suppression), not clinically sedated ICU patients. The note appears in the table footnote, the results text, and the Discussion. We have added a direct statement: 'Clinical validation in true ICU sedation populations — where patients are mechanically ventilated and fully unconscious — is a required and distinct future validation step before any claims can be made for that population.' The low-expresser result (37.6% improvement) is now contextualized as pertaining to healthy volunteers with suppressed voluntary expression, not pathologically absent expression.

Minor Comment 7 — Data policy / PLOS exception

PLOS Data policy requires all data be made available with rare exceptions. In the data availability statement, authors make available code and processed features but cannot make available facial images/video due to protected health information (PHI) and IRB concerns, which I believe to be reasonable and qualifies for the rare exception.

Thank you for this assessment. We have implemented a three-tier data availability structure as described in our response to Journal Requirement 6: (1) code/models — fully public; (2) anonymized processed features and metadata — deposited in Supporting Information (S2–S3 Tables); (3) raw video — IRB-exemption request submitted. The Data Availability Statement in the manuscript has been updated to make these three tiers explicit and clearly distinguishable.

REVIEWER #2 — Pei-Chun Lin

Comment 1 — Introduction and Related Work: overly long, objectives unclear

The Introduction section contains overly lengthy paragraphs; it would be beneficial to make this section more concise. In addition, the research objectives should be stated more clearly to better highlight the purpose and contribution of the study. A similar issue is observed in the Related Work section, where the descriptions are also excessively long and could be streamlined.

Fully addressed — see also Reviewer 1 Major Comments 1–3. The Introduction has been condensed to approximately 1.5 pages. Research objectives are now explicitly stated in the final paragraph of the Introduction as three testable hypotheses (H1: thermal pain patterns exist; H2: multimodal fusion outperforms single modality; H3: temporal dynamics capture progression with thermal precedence) and four numbered contributions. The Related Work section has been removed as a standalone section; its content has been redistributed into the Methods (prior work context) and Discussion (comparison to prior work sub-section).

Comment 2 — Why is MAE used as primary metric rather than accuracy?

In Table 1, it appears that previous studies have primarily reported performance using accuracy as the evaluation metric. The authors should clarify why MAE is used as the primary metric in this manuscript.

A dedicated justification paragraph has been added to Section 4.2 (Evaluation Metrics). Key arguments:

- Task framing: Our system estimates pain as a continuous intensity value on the 0–10 NRS scale (regression), not a categorical label. MAE is the natural primary metric for regression tasks.
- Clinical interpretability: An MAE of 0.87 corresponds directly to less than 1 NRS point average error, which is below the minimal clinically important difference of 1.5 points commonly used in pain trials (Herr et al. 2019). Accuracy percentage is harder to translate into clinical meaning.
- Prior regression literature: MAE is the standard primary metric in continuous pain regression work (Zhou et al. 2021; Gkikas et al. 2024; Khan et al. 2025). Studies reporting accuracy use coarse categorical labels (e.g., none/mild/severe) which are not directly comparable to regression outcomes.
- We retain 3-class accuracy as a secondary metric to facilitate comparison with classification-framed prior work, and it is now clearly labeled as secondary throughout.

Comment 3 — Dataset description unclear; final dataset size not specified

The description of the Datasets should also be presented more clearly, particularly regarding the final dataset size used in the study.

Section 4.1 (Datasets) has been revised to open with a dedicated summary paragraph explicitly stating all final dataset sizes:

- Dataset 1: 50 subjects, 312 sessions, 87.3 hours, 9.46 million frames at 30 fps, frame-level NRS labels sampled at 1 Hz.
- Dataset 2: 30 subjects, 17.7 hours, 1.92 million frames, NRS labels every 2 minutes.
- Combined: 80 subjects, 105 hours, 11.38 million frames total.
- Table 1 (Dataset Statistics) now also includes per-fold training and test frame counts (~7.57M / ~1.89M for Dataset 1; ~1.54M / ~0.38M for Dataset 2), making the 5-fold split completely transparent.

Comment 4 — Report error ranges (std or CI) for 5-fold CV results in Table 3

Since the study employs 5-fold cross-validation, it is recommended to report each evaluation metric together with its error range (e.g., standard deviation or confidence interval) to provide a more comprehensive assessment of model performance.

All tables reporting model performance (Tables 2–5 in the revised manuscript) now include mean±std across the 5 folds for all reported metrics (MAE, PCC, ICC, 3-class Accuracy). For the primary metric MAE of the final model, 95% confidence intervals are additionally reported, computed via bootstrap resampling (B=1000) from the pooled test-set predictions across folds.

All bar charts in the figures have been updated to display 95% CI error bars. Table footnotes now explicitly state: 'All values: mean±std across 5 cross-validation folds. 95% CI for CSAF+Transformer primary metric computed via bootstrap (B=1000).'

Comment 5 — Clarify explanations for all figures and tables

The explanations for all figures and tables should also be clarified to improve readability and interpretation.

All figure captions and table notes have been revised to be fully self-contained and interpretable without reference to the main text. Specific changes:

- All abbreviations are now defined within each caption/footnote on first use.
- Arrow symbols (↓ lower is better, ↑ higher is better) are defined in table column headers or footnotes.
- Statistical significance notation (*** $p<0.001$, ** $p<0.01$) is explained in every table footnote.
- Bonferroni correction details (number of comparisons, α threshold) are stated in table footnotes.
- The cross-validation fold averaging procedure is stated explicitly: 'mean±std across 5 stratified cross-validation folds.'
- Figure captions now describe each panel (a), (b), (c)... individually rather than collectively.
- The human expert comparison figure caption now explicitly states the information asymmetry caveat.

We thank both reviewers and the editorial team once more for their rigorous and constructive evaluation. We believe the revised manuscript is substantially improved and hope it now fully meets the publication standards of *PLOS Digital Health*. We look forward to your decision.

Corresponding author: Sami Naouali (salnawali@kfu.edu.sa)
