# Supplementary material for: Cross-spectral fusion of thermal and RGB imaging for objective pain estimation
Source: PLOS Digit Health. 2026 May 15;5(5):e0001424. doi: 10.1371/journal.pdig.0001424 (PMC13178875; doi:10.1371/journal.pdig.0001424)
Supplement: Attachment — Submitted filename: Response_to_Reviewers_auresp_2.pdf [file pdig.0001424.s004.pdf]

RESPONSE TO REVIEWERS

Second Revision · PDIG-D-26-00203R2

Cross-Spectral Fusion of Thermal and RGB Imaging for Objective Pain Estimation

Oussama El Othmani · Sami Naouali

PLOS Digital Health · April 2026

General Response

We thank the Academic Editor (Dr. Jia-Lang Xu) and both reviewers for their constructive evaluation of our revised manuscript. We are grateful to Reviewer 1 (Philip Chung) for confirming that all prior comments have been satisfactorily addressed, and to Reviewer 2 (Pei-Chun Lin) for the additional comments that have helped us further improve the work.

Below we address each remaining point in full. All manuscript changes are highlighted in blue in the Revised Manuscript with Track Changes file. The exact LaTeX insertions are provided in the LaTeX_Patches_Clean.tex and LaTeX_Patches_Tracked.tex supplementary files.

Reviewer 1 (Philip Chung)

Overall status: Reviewer 1 confirmed that all prior comments have been addressed and the manuscript is substantially improved. One remaining comment was raised, detailed below.

Comment R1-1 · Figures 7 & 8 — Spatial context for heatmaps

> *"It is unclear how to interpret spatial attention maps without facial image context. The captions claim attention is focused on specific parts of the face, but looking at the heatmaps alone, I cannot tell if there is a face in the heat map and where the facial features would be located. To assist the reader in interpreting these figures while protecting identities of original participants, I recommend superimposing a representative "average" sketched face diagram on the heatmap. This would allow the reader to link the heatmaps to specific facial regions."*

Response:

We thank Reviewer 1 for this clear and practical suggestion. We have fully implemented it in both affected figures.

Figure 7 (Spatial Attention Maps): A representative average face schematic — computed from average face geometry, with no participant images or photographs used — has been overlaid on each of the six panels as a white line drawing (face oval, eyes, brows, nose, mouth, ears). For the Cheek and Perioral RGB attention panels, and the Periorbital and Forehead thermal attention panels, a dashed yellow bounding box additionally outlines the specific anatomical region of interest, directly linking the heatmap to the facial region named in the caption.

Figure 8 (Thermal Patterns): The same face schematic overlay and dashed yellow ROI boxes have been applied to all four thermal pattern panels (Nasal Cooling, Periorbital Warming, Forehead Cooling, Thermal Asymmetry). The ROI box is positioned at the anatomically correct location for each pattern (nasal region for Pattern A, periorbital for Pattern B, forehead for Pattern C).

Caption expansion: Both figure captions have been substantially expanded to include panel-by-panel anatomical interpretation (see Comments R2-2 and R2-3 for detail). The combination of the face sketch, the ROI box, and the expanded caption now gives readers three complementary sources of spatial context.

A legend note at the bottom of each figure explicitly states: "White outline = representative average face schematic (no participant data shown)" and "Dashed yellow box = region of interest highlighted per panel", pre-empting any potential confusion about the source of the overlay.

> ▶ **Changes made:** *Figure 7 and Figure 8 regenerated with white face schematic overlay and dashed yellow ROI boxes. Both figure captions expanded (see R2-2/R2-3). LaTeX filename update (Patch TC-7).*

Reviewer 2 (Pei-Chun Lin)

Reviewer 2 raised four comments requiring further revision. We address each in detail below.

Comment R2-1 · Introduction too brief

> *"The Introduction section remains too brief. Please expand it to better highlight the novelty and significance of this study."*

Response:

We agree that the Introduction, while restructured in R1 to improve flow, did not sufficiently articulate the novelty and clinical significance of the cross-spectral fusion approach. We have added a new dedicated paragraph that addresses three dimensions of significance:

- The specific unmet clinical need: automated RGB-only systems fail in precisely the patient populations (low expressers, sedated, cognitively impaired, culturally stoic) where objective pain measurement matters most;
- The physiological motivation for bimodal fusion: RGB captures the voluntary-motor component of pain (behavioral expression); thermal captures the involuntary-autonomic component (vasoconstriction, metabolic heat). These are distinct physiological channels operating on different neural pathways and different timescales;
- Explicit linkage to results: the new paragraph concludes by pointing directly to the largest demonstrated benefits — 37.6% improvement for low expressers and 32.1% for sedated-controlled subjects — making the clinical significance concrete.

The new paragraph is positioned after the thermal imaging motivation paragraph (which establishes why thermal is physiologically motivated) and before the hypotheses paragraph (which formalises the research questions), providing a logical bridge between clinical background and formal hypotheses.

> ▶ **Changes made:** *New paragraph inserted in Introduction (after thermal imaging motivation, before hypotheses). Discusses the two distinct physiological channels, the clinical gap for suppressed-expression populations, and links explicitly to results. See Patch TC-1 in LaTeX_Patches_Tracked.tex.*

Comment R2-2 · Table and figure descriptions too concise

> *"The descriptions of the tables and figures are overly concise and should be elaborated to improve clarity and interpretability."*

Response:

We have expanded captions for all key figures and tables. The changes are as follows:

Figure/Table	What was added	Patch ref.
**Table 2 (Baseline)**	Added interpretive sentence: 29.3% improvement exceeds cumulative CNN→LSTM→Transformer gain (~10–17%), contextualising modality augmentation vs architectural refinement.	TC-5
**Figure 2 (Performance)**	Added: what each axis represents, how to read error bars, and explicit main finding sentence.	(inline)
**Figure 3 (Intensity)**	Added: explicit description of the monotonically increasing benefit pattern and what it implies physiologically (expression ceiling effect + stronger autonomic signals).	(inline)

Figure/Table	What was added	Patch ref.
**Figure 7 (Attention)**	Fully rewritten with panel-by-panel anatomical analysis, AU linkage, key insight on spatial complementarity. See R2-3 for detail.	TC-2
**Figure 9 (Populations)**	Added: explicit interpretation of the low-expressor and dark-skin-tone results and what they imply for clinical utility.	(inline)
**Figure 8 (Thermal patterns)**	Fully rewritten with panel-by-panel physiological mechanistic interpretation. See R2-3 for detail.	TC-4

> ► **Changes made:** *Captions expanded for Table 2, Figures 2, 3, 7, 8, and 9. See Patches TC-2, TC-4, TC-5 in LaTeX_Patches_Tracked.tex and corresponding sections in LaTeX_Patches_Clean.tex.*

Comment R2-3 · Figure 7 (attention maps) analysis insufficient

> *"Figure 7 is a critical component of this manuscript. Please strengthen the analysis in this section and identify the common patterns or key insights derived from these visualizations."*

Response:

We have made four improvements targeting this figure (now renumbered Figure 7):

(1) Face schematic overlay: As described in R1-1, the face sketch and ROI boxes now provide immediate spatial context, removing the ambiguity Reviewer 1 identified and enabling readers to directly connect heatmap activations to anatomy.

(2) Expanded figure caption — panel by panel: The caption has been rewritten from 3 generic sentences to a comprehensive panel-by-panel analysis covering: spatial localisation (where on the face), anatomical identity (which structures), physiological mechanism (why that region activates), and AU linkage (RGB panels). For example, the Perioral RGB panel caption now explicitly states that the high activation at the mouth reflects AU9/10 (levator labii superioris and lip corner depressor) — canonical FACS pain action units per Prkachin & Solomon (2008).

(3) Key insight identified explicitly: The caption now closes with a "Key insight" statement: RGB and thermal attention maps have largely non-overlapping spatial profiles — RGB concentrates on the lower face (voluntary expressive regions) while thermal targets the upper face and nasal area (vascular-autonomic regions). This spatial complementarity is identified as the mechanistic basis for the multimodal improvement, validating both H1 and H2.

(4) Section 4.7 in-text expansion: The in-text analysis in Section 4.7 has been extended from one sentence to a full paragraph that: (a) describes both spatial patterns, (b) identifies the non-overlapping nature as the key observation, and (c) explains why this complementarity means the thermal advantage is largest precisely when behavioral expression is suppressed.

> ► **Changes made:** *Figure 7 updated (face overlay + ROI boxes). Caption fully rewritten with panel-by-panel analysis and explicit "Key insight" statement. Section 4.7 in-text expanded to paragraph. See Patches TC-2 and TC-3 in LaTeX_Patches_Tracked.tex.*

Comment R2-4 · Discussion insufficient

> *"The Discussion section is still insufficient. Please expand it by elaborating on the study findings and providing a more thorough comparison with existing literature."*

Response:

We have substantially expanded the Discussion from approximately 600 words to approximately 1,100 words. The revision adds three specific elements Reviewer 2 requested:

1. Thorough comparison with existing literature

We have added explicit quantitative comparisons to the most relevant recent work:

- Gkikas et al. (2024): strong binary accuracy (82.7%) fusing RGB+heart rate, but no continuous NRS regression, no low-expressor analysis, no clinical data. Our work addresses all three gaps.

- Khan et al. (2025) systematic review: specifically identifies non-contact thermal multimodal fusion as an open gap in the field. Our work is the first published study to fill this gap.
- Thiam et al. (2019) and Lin et al. (2022): wearable multimodal approaches with which we directly compare. Our non-contact system achieves MAE=0.87 vs 1.04 for RGB+wearable physio, without the deployment barriers of skin-contact sensors.
- Human expert comparison: we now discuss the information asymmetry caveat (nurses had clinical context) and note that our ICC of 0.84 exceeds the 0.55–0.75 ICC of standard observational scales (Herr et al. 2019), quantifying the reliability improvement.

2. New subsection: Physiological Interpretation of Thermal Patterns

A new "Physiological Interpretation of Thermal Patterns" subsection has been added to the Discussion, providing mechanistic explanations for all four discovered patterns:

- Nasal cooling (42%): dense sympathetic innervation + limited collateral circulation make nasal tip the most sensitive thermal index of sympathetic tone. Consistent with CPT literature (Mourot 2009) and chronic pain thermography (Jeyaraman 2025).
- Periorbital warming (38%): metabolic heat from orbicularis oculi contraction (AU6/7), directly connecting thermal to FACS pain AUs. Explains why both modalities attend to the orbital region for different reasons.
- Forehead cooling (28%): global sympathetic vasoconstriction during acute stress, tends to co-occur with nasal cooling.
- Thermal asymmetry (18%): possible hemispheric lateralization, with clinical precedent in neuropathic pain thermography (Schenone 2019).

Critically, the new subsection identifies that patients with predominantly nasal-cooling patterns are "autonomic responders" — the subgroup with the largest benefit (low expressers, 37.6%) and the greatest current clinical unmet need.

3. Extended limitations

The limitations subsection has been expanded to add:

- Cultural and ethnic diversity: Fitzpatrick skin types I–VI were represented but race/ethnicity was not collected. Future work should include self-reported ethnicity and assess performance across groups differing in pain expressivity norms.
- Hardware accessibility: FLIR A655sc cost (~$10,000) as a barrier, with consumer-grade validation (~$500, 0.1–0.2°C sensitivity) identified as a priority future direction.
- Sedated-population scope: explicit clarification that the "sedated" subgroup is pharmacologically reduced expression in a controlled lab, not ICU ventilated patients.

> ► **Changes made:** *Discussion expanded from ~600 to ~1,100 words. New explicit comparisons to Gkikas 2024, Khan 2025, Thiam 2019, Lin 2022. New "Physiological Interpretation of Thermal Patterns" subsection. Limitations extended with cultural diversity and hardware cost. See Patch TC-6 in LaTeX_Patches_Tracked.tex.*

Summary of All Changes for R2

Comment	Nature of change	Manuscript location	Patch
**R1-1**	Face schematic overlay + ROI dashed boxes on Figs 5 & 11; captions expanded	Figs 7, 8 (PDFs); captions	**TC-2, TC-4, TC-7**
**R2-1**	New Introduction paragraph: clinical gap, dual physiological channels, target populations	§1 Introduction	**TC-1**
**R2-2**	Captions expanded: Table 2, Figs 2, 3, 7, 8, 9	Throughout	**TC-2, TC-4, TC-5**

Comment	Nature of change	Manuscript location	Patch
**R2-3**	Fig 7 overlay + caption panel analysis + "Key insight" + §4.7 in-text expansion	Fig 5, §4.7	**TC-2, TC-3**
**R2-4**	Discussion: lit comparison (4 papers), new physiology subsection, extended limitations	§5 Discussion	**TC-6**

We believe the revised manuscript now fully addresses all remaining reviewer concerns. The face schematic overlay on Figures 7 and 8 directly resolves the spatial interpretability issue raised by Reviewer 1. The expanded Introduction, figure captions, in-text analysis, and Discussion respond comprehensively to Reviewer 2's four comments on clarity, depth, figure analysis, and literature comparison.

We are grateful for the reviewers' thorough engagement with this work and look forward to the editor's decision.

Sincerely,

Oussama El Othmani · Sami Naouali

Computer Science Dept., Military Academy of Fondouk Jedid, Tunisia | King Faisal University, Al Ahsa, Saudi Arabia
